# Draft Genome Sequence of *Neisseria gonorrhoeae* Sequence Type 1407, a Multidrug-Resistant Clinical Isolate

**DOI:** 10.1128/genomeA.00903-15

**Published:** 2015-08-13

**Authors:** A. Anselmo, A. Ciammaruconi, A. Carannante, A. Neri, C. Fazio, A. Fortunato, A. M. Palozzi, P. Vacca, S. Fillo, F. Lista, P. Stefanelli

**Affiliations:** a Army Medical and Veterinary Research Center, Rome, Italy; b Department of Infectious, Parasitic and Immuno-Mediated Diseases, Istituto Superiore di Sanità, Rome, Italy

## Abstract

Gonorrhea may become untreatable due to the spread of resistant or multidrug-resistant strains. Cefixime-resistant gonococci belonging to sequence type 1407 have been described worldwide. We report the genome sequence of *Neisseria gonorrhoeae* strain G2891, a multidrug-resistant isolate of sequence type 1407, collected in Italy in 2013.

## GENOME ANNOUNCEMENT

The emergence and spread of antimicrobial resistance in *Neisseria gonorrhoeae* requires a deeper knowledge of the genetic basis of resistance. The use of genomes for surveillance purposes could be considered a useful strategy to improve public health microbiology (1).

We announce the draft genome sequence of *N. gonorrhoeae* G2891, collected in 2013 from a heterosexual man of 50 years with urethritis. The isolate was resistant to cefixime (MIC, 0.19 mg/liter), ciprofloxacin (MIC, 32 mg/liter), and azithromycin (MIC, 1.5 mg/liter).

The isolate belonged to ST1407 according to *Neisseria gonorrhoeae* multi-antigen sequence typing (NG-MAST); this sequence type, together with closely related sequence types, defines the genogroup (G) 1407, which is mainly associated with resistance to extended-spectrum cephalosporins (2, 3).

DNA was extracted using the QiAmp minikit (Qiagen, Hilden, Germany) from an overnight culture grown on a Thayer Martin plus 1% IsoVitalex (Oxoid, Hampshire, United Kingdom) agar plate at 37°C. Antimicrobial susceptibility was obtained by *E* test (bioMérieux, Sweden) according to the manufacturer’s instructions. The clinical breakpoints were those indicated by the European Committee on Antimicrobial Susceptibility testing (EUCAST, version 5.0, 2015) (4).

The *porB* and *tbpB* alleles were assigned at the NG-MAST website (http://www.ng-mast.net). Whole-genome sequencing was performed using both the MiSeq and the NextSeq 500 Illumina platforms. The MiSeq run generated 2,451,407 paired-end reads (300 × 2 bp), while the NextSeq 500 produced 4,078,929 paired-end reads (150 × 2 bp). Reads were trimmed to keep high-quality bases (*Q* score >25): 2,113,645 and 2,796,663 paired-end reads, respectively, were further analyzed (coverage ~600×). For both sets of reads, *de novo* assembly was performed using ABySS version 1.3.5 (5) and Velvet version 1.2.10 (6). To improve the contiguity and the accuracy of the assembly, contigs resulting from different analysis were integrated with CISA (7). The final assembly was composed of 132 contigs (>500 nucleotides), and the draft genome sequence was 2,149,953 bp long.

The 132 contigs were submitted to the NCBI whole-genome shotgun (WGS) submission portal (https://submit.ncbi.nlm.nih.gov/subs/wgs), and the sequence was annotated using the NCBI Prokaryotic Genome Automatic Annotation Pipeline (PGAAP) (http://www.ncbi.nlm.nih.gov/genome/annotation_prok).

Of note, *N. gonorrhoeae* G2891 shared the *penA* mosaic allele XXXIV (GenBank accession number GU723422.1); this *penA* allele was described for the first time in San Francisco in 2008 (8) and has been commonly associated with cefixime resistance (9). Moreover, the deletion A in the promoter region and the amino acid substitution H105Y in the *mtrR* gene, the amino acid substitution L421P in the *ponA* gene, and the amino acid substitutions G120K and A121N in the *porB1b* gene were identified. By multilocus sequence typing (MLST), the isolate belonged to ST1901.

### Nucleotide sequence accession number.

This whole-genome shotgun project has been deposited in DDBJ/GenBank under the accession number LDOB00000000**.**
